# The dual S1PR1/S1PR5 drug BAF312 (Siponimod) attenuates demyelination in organotypic slice cultures

**DOI:** 10.1186/s12974-016-0494-x

**Published:** 2016-02-08

**Authors:** Catherine O’Sullivan, Anna Schubart, Anis K. Mir, Kumlesh K. Dev

**Affiliations:** Drug Development, School of Medicine, Trinity College, Dublin, Ireland; Novartis Institutes for BioMedical Research, Novartis Pharma AG, Basel, Switzerland

**Keywords:** Sphingosine-1 phosphate receptors (S1PRs), Siponimod (BAF312), Aastrocytes, Demyelination, Krabbe disease

## Abstract

**Background:**

BAF312 (Siponimod) is a dual agonist at the sphingosine-1 phosphate receptors, S1PR1 and S1PR5. This drug is currently undergoing clinical trials for the treatment of secondary progressive multiple sclerosis (MS). Here, we investigated the effects of BAF312 on isolated astrocyte and microglia cultures as well as in slice culture models of demyelination.

**Methods:**

Mouse and human astrocytes were treated with S1PR modulators and changes in the levels of pERK, pAkt, and calcium signalling as well as S1PR1 internalization and cytokine levels was investigated using Western blotting, immunochemistry, ELISA and confocal microscopy. Organotypic slice cultures were prepared from the cerebellum of 10-day-old mice and treated with lysophosphatidylcholine (LPC), psychosine and/or S1PR modulators, and changes in myelination states were measured by fluorescence of myelin basic protein and neurofilament H.

**Results:**

BAF312 treatment of human and mouse astrocytes activated pERK, pAKT and Ca^2+^ signalling as well as inducing S1PR1 internalization. Notably, activation of S1PR1 increased pERK and pAKT in mouse astrocytes while both S1PR1 and S1PR3 equally increased pERK and pAKT in human astrocytes, suggesting that the coupling of S1PR1 and S1PR3 to pERK and pAKT differ in mouse and human astrocytes. We also observed that BAF312 moderately attenuated lipopolysaccharide (LPS)- or TNFα/IL17-induced levels of IL6 in both astrocyte and microglia cell cultures. In organotypic slice cultures, BAF312 reduced LPC-induced levels of IL6 and attenuated LPC-mediated demyelination. We have shown previously that the toxic lipid metabolite psychosine induces demyelination in organotypic slice cultures, without altering the levels of cytokines, such as IL6. Importantly, psychosine-induced demyelination was also attenuated by BAF312.

**Conclusions:**

Overall, this study suggests that BAF312 can modulate glial cell function and attenuate demyelination, highlighting this drug as a further potential therapy in demyelinating disorders, beyond MS.

## Background

The family of spingosine-1-phosphate receptors (S1PR) have been rapidly gaining attention as important mediators of many cellular processes, including cell differentiation, migration, survival, angiogenesis, calcium homeostasis, inflammation and immunity [1]. These receptors are G-protein coupled and are known targets for the drug Gilenya® (pFTY720), an oral therapy used in patients with multiple sclerosis (MS) [2]. The phosphorylated version of FTY720 (pFTY720) acts on four of the five S1PRs (S1PR1, S1PR3, S1PR4 and S1PR5) and acts as a functional antagonist at the S1PR1 subtype by causing S1PR1 internalisation, thereby inhibiting lymphocyte egress from lymph nodes to the periphery and central nervous system (CNS) [3]. FTY720 can also cross the blood-brain barrier, where it is then phosphorylated and likely regulates neuronal and glial cells [4, 5]. S1PR expression is cell-type dependent and these receptors are known to play a role in, for example, astrocyte migration, oligodendrocyte myelination state and neurite outgrowth and neurogenesis [5–9].

Since the development of pFTY720 and its demonstrated clinical efficacy in MS, there have been ongoing efforts to develop more selective S1PR agonists and antagonists [10]. These compounds have focused primarily on creating selectivity for S1PR1 and/or S1PR5, with limited activity for S1PR3. Reasons for this, in most part, are due to suggestions that S1PR1 regulates inflammatory response [11], that S1PR1 and S1PR5 can promote myelination state [4, 12–14], but that S1PR3 activation induces bradycardia [10, 15]. Siponimod (BAF312) ((*E*)-1- (4-(1-(((4-cyclohexyl-3-(trifluoromethyl)benzyl)oxy)imino)ethyl)-2-ethylbenzyl)azetidine-3-carb oxylic acid)) is a S1PR1/S1PR5 dual agonist that has been developed by modification of the hydrophobic alkyl chain in FTY720 and replacement of the *n*-octyl moiety with a substituted benzyloxy oxime moiety [10]. Further replacement of an amino phosphate moiety of FTY720 by amino carboxylic acids has provided BAF312 with shorter elimination half-lives in vivo and with the added benefit of developing a ‘non’ pro-drug [16]. In rat models of experimental autoimmune encephalomyelitis (EAE), BAF312 suppresses preclinical symptoms [16]. Interestingly, however, despite sparing S1PR3 activity, BAF312 still causes bradycardia in humans; however, this can be mitigated using a novel dose titration scheme (up to 2–10 mg over 9/10 days) [10, 16, 17]. BAF312 has successfully undergone phase II clinical trials for relapsing remitting MS warranting further phase III trials [18].

Here, we investigate the effects of BAF312 on S1PR trafficking, signalling and pro-inflammatory cytokine levels in astrocytes and microglia, as well as its effects on demyelination in organotypic slices cultures. We also demonstrate the effects of BAF312 in a demyelination slice culture model of globoid cell leukodystrophy (Krabbe disease, KD) using the toxic lipid metabolite psychosine.

## Methods

### Glial and cerebellar slice cultures

Human and mouse astrocytes were cultured as we have described before [19, 20]. Microglia were prepared as mixed glia cultures as described [19, 20] and grown in DMEM/F12 (Fisher) supplemented with 10 % heat inactivated foetal bovine serum (FBS) (Labtech, FB-1090), 1 % penicillin/streptomycin (Sigma, P4333) and M-CSF (20 ng/ml) and GM-CSF (10 ng/ml). After 12 days in culture, the microglia were separated from astrocytes by placing them on a rotating shaker for 2–3 h at room temperature. The media containing the microglia was then centrifuged for 3 min at 2000 rpm. The supernatant was discarded and the resultant pellet resuspended in 1 ml of warmed media. Cells were counted using Bright line and plated (1 × 10^5^ cells/ml) in 24-well plates and incubated for 24 h before the addition of warmed supplemented DMEM. In all cases, before treatments, the cells were serum starved for 3–4 h by incubating in serum-free DMEM/F12 at 37 °C and 5 % CO_2_. Specific treatment details are indicated in the figure legends. Organotypic cerebellar slice cultures were prepared exactly as we have described previously [9, 21]. In brief, tissue isolated from postnatal day 10 (P10) C57BL/6 mice and 400 μm parasagittal slices of cerebellum were grown on cell culture inserts (five to six slices each) (Millicell PICMORG50 Millipore). Slices were cultured using an interface method with 1 ml of medium per 35 mm well. For the first 3 days in vitro (DIV), the slices were grown in serum-based medium (50 % Opti-Mem (Invitrogen), 25 % Hanks’ buffered salt solution (HBSS) (Gibco), 25 % heat-inactivated horse serum and supplemented with 2 mM Glutamax, 28 mM D-glucose, 100 U/mL penicillin/streptomycin (Sigma) and 25 mM HEPES, (Sigma) at 35.5 °C and 5 % CO_2_. After three DIV, the slices were transferred to serum-free medium (98 % Neurobasal-A and 2 % B-27 (Invitrogen), supplemented with 2 mM Glutamax, 28 mM D-glucose, 100 U/mL penicillin/streptomycin and 25 mM HEPES). Demyelination was induced at 12 DIV and examined at 14 DIV. All tissue was isolated in accordance with EU guidelines and protocols approved by the Trinity College Dublin ethics committee.

### Biochemical analysis

For cytokine analysis, supernatants from cell culture were removed and examined using ELISA for IL6 (DY406) according to the manufacturer’s instructions (R&D systems) and exactly as we have described before [21]. For Western blotting, astrocytes were scraped from the culture plate and suspended in PTxE buffer (PBS, 1 % Triton-X, 1 mM EDTA). Samples were denatured and electrophoresis carried out on 10 % SDS-polyacrylamide gels exactly as we have previously reported [21]. Primary antibodies used were anti-pERK (Millipore, 05-797R), anti-pAKT (Cell Signalling, S473), anti-ERK 1/2 (Millipore 05-481) and anti-actin (Abcam, ab3280). Secondary antibody used was HRP conjugated mouse (Sigma, A8924) or rabbit (GE Healthcare, NA934).

### Calcium signalling

Calcium signalling was performed as previously described [19, 22]. Briefly, cells were grown until ∼80 % confluency on glass-bottomed FluoroDishes (World Precision Instruments, Sarasota, FL, USA). Cells were serum starved for 3 h and pre-treated for 1 h with S1PR1 antagonist (NIBR-0213) [23] or left in serum-free media. The cells were washed with 1 ml 37 °C HBSS (Invitrogen) supplemented with 20 mM HEPES buffer (Invitrogen) and 5.5 mM glucose (Sigma Aldrich). Cells were then loaded with 2 μM Fluo-8 AM (Invitrogen) in supplemented 37 °C HBSS for 40 min at 37 °C and 5 % CO2. Fluo-8 AM dye was removed and cells were washed with 37 °C supplemented HBSS. Next, cells were left to rest in 1 ml supplemented HBSS at room temperature in the dark for 20 min. Calcium responses were recorded using an Olympus FV1000 Confocal Microscope with ×20 lens. For analysis, images were obtained at a rate of 1 frame/2 s for a total of 250 s. Images were then analysed using the Olympus Fluoview viewer software. Fluorescence was normalised to mean baseline fluorescence (0–30 s) (Δ*F*/*F*0). GraphPad Prism 4 software was used to generate calcium response traces presented as Δ*F*/*F*0 over time.

### Immunocytochemistry

Post-treatment mouse astrocytes were washed with PBS (Sigma Aldrich, UK) and fixed with ice-cold PFA buffer (3.7 % formaldehyde in PBS, pH 7.4). Cells were washed in PBS, permeabilised with PTx buffer (0.1 % Triton X-100 in PBS, pH 7.4) and washed again with PBS. Non-specific binding was reduced by incubating cells for 1 h at room temperature in TwB buffer (PBS supplemented with 0.1 % Tween 20, 1 % BSA). For all antibody incubation steps, TwB buffer (PBS supplemented with 0.05 % Tween-20 and 0.5 % BSA) was used. The cells were incubated overnight at 4 °C with primary antibodies. For S1PR1 staining, cells were incubated with biotinylated anti-rabbit (Vector, BA-1000) for 2 h. The cells were then washed and incubated with Avidin-Alexa 488 coupled secondary antibody (1/1000 dilution: Invitrogen, S-11223) and DyLight 549 conjugated anti-mouse (1/1000 dilution: Jackson Immunoresearch) for a further 45 min. Cells were washed twice with PBS again and incubated with Hoechst in PBS for 10 min. Finally, the cells were washed and coverslips mounted onto a drop of Vectorshield on a glass slide and sealed with nail polish. The cells were visualised with an Olympus FV1000 scanning confocal. For organotypic cerebellar slice cultures, immunostaining was performed as we have described previously [9]. Confocal images were captured using a LSM 510 Meta microscope at ×10 or ×20 magnification. These resulting images were analysed using ImageJ software. A total number of five to six slices were used per condition, and the fluorescence of each cerebellar slice was captured using five to six independent regions of interest (ROI). The ROI were selected randomly to cover the whole slice and the mean fluorescence was calculated using a total of 25–36 independent ROI observations, for each independent experiment. Primary antibodies used were mouse anti-Vimentin (1/1000 dilution: Santa Cruz sc-373717), rabbit anti-S1PR1 (1/500 dilution: Santa Cruz sc-25489), rabbit anti-myelin basic protein (MBP) (1/1000 dilution: Abcam ab40390) and chicken anti-neurofilament H (NFH) (1/1000 dilution: Millipore, MAB5539). Secondary antibodies used were DyLight 549 conjugated anti-mouse (1/1000: Jackson Immunoresearch), Alexa Fluor 488 anti-rabbit (1/1000: Life Technologies A11008) and Alexa Fluor 633 anti-chicken (1/1000: Life Technologies A21103).

### Statistical analysis

All statistical analysis was performed using Prism 5 GraphPad Software package. A one-way ANOVA with Newman-Keuls post hoc test was used to compare groups. The significance levels (or alpha levels) were set at *p* < 0.05*, *p* < 0.01** and *p* < 0.001***.

## Results

### Activation of S1PR1/S1PR5 promotes pERK and pAKT signalling in mouse and human astrocytes

**Fig. 1:**
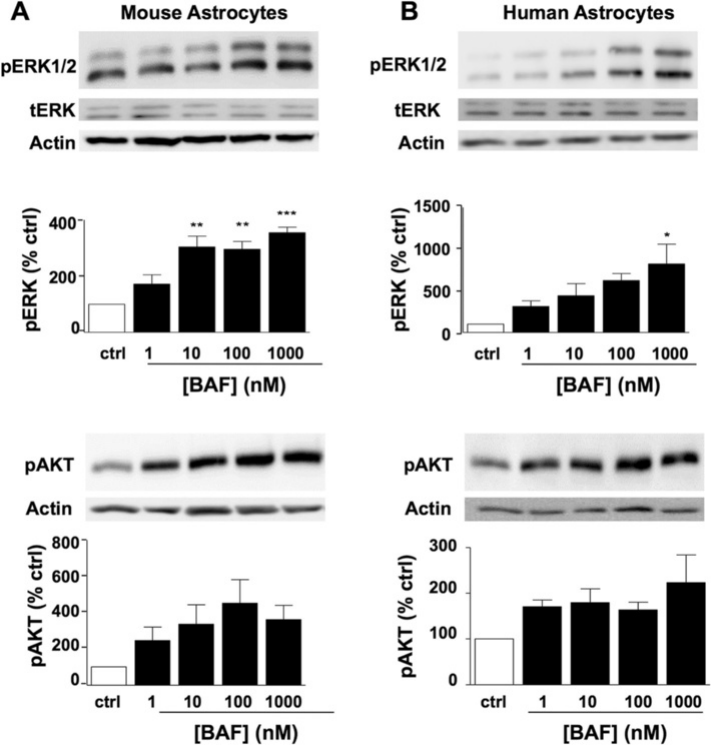
Agonism of S1PR1/5 promotes pERK and pAkt signalling in mouse and human astrocytes. BAF312 at concentrations shown induced pERK after 10 min (*n* = 3) and pAKT after 30 min treatments (*n* = 4) **a** in mouse astrocytes and **b** in human astrocytes. Data presented as ±SEM (*n* = 3-4), one-way ANOVA and Newman-Keuls multiple comparison post-test. *p < 0.05, **p < 0.01, ***p < 0.001

The Ras/Raf/MEK/ERK and PI3K/PTEN/AKT signalling cascades are key signalling pathways involved in the regulation of cell survival and proliferation of numerous cell types. Activation of the S1PRs is known to induce potent ERK phosphorylation in astrocytes [24] and AKT phosphorylation in various mammalian cells [25, 26]. Induction of pERK is known to be a transient response with maximal induction of signalling seen between 10 and 30 min of treatment [24]. Here, we investigated the effect of the S1PR1/S1PR5 agonist, BAF312, on ERK and AKT phosphorylation in human and mouse astrocytes. Cultured human and mouse astrocytes were serum starved for 4 h and then treated with increasing concentrations of BAF312 (1 nM, 10 nM, 100 nM and 1 μM) for 10 min (pERK) or 30 min (pAKT) and the samples prepared for Western blotting. As expected, the treatment of astrocytes with BAF312 for 10 and 30 min induced ERK and AKT phosphorylation, respectively, in a concentration-dependent manner in both mouse and human astrocytes (Fig. 1). These data suggest that a lack of S1PR3 activity does not impede the ability of BAF312 to regulate ERK and AKT signalling pathways.

### Differential roles for S1PR1 and S1PR3 in pERK signalling in mouse and human astrocytes

**Fig. 2:**
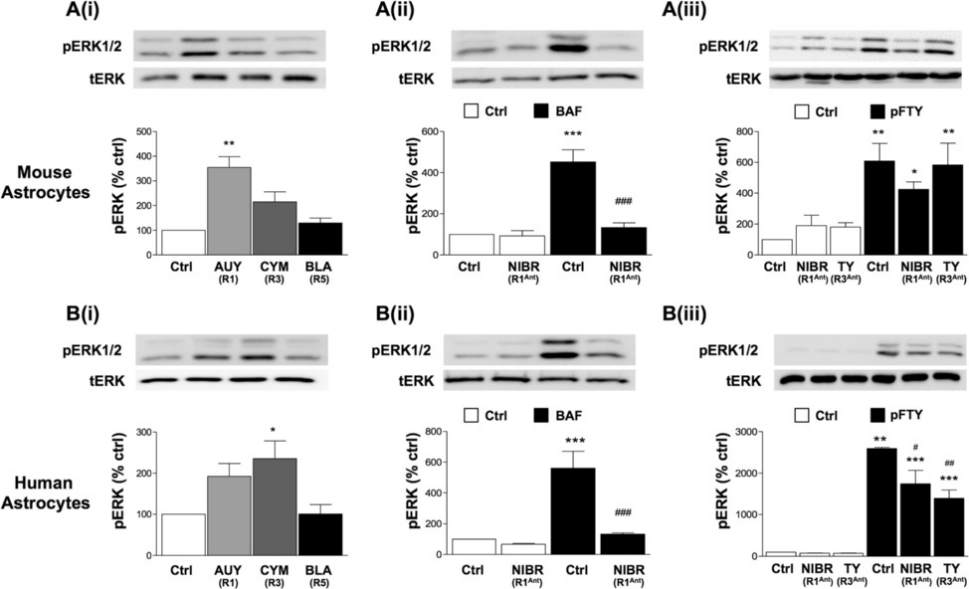
Differential roles for S1PR1 and S1PR3 in pERK signalling in mouse and human astrocytes. Astrocytes were serum starved for 4 h before all treatments. All treatment with agonists were for 10 min and pretreatment with antagonists were at 1 μM for 1 h. **Ai** AUY954 (S1PR1 agonist, 1 μM), but not CYM5541 (S1PR3 agonist, 1 μM) or UC-42-WP04 (S1PR5 agonist, 1 μM) induced pERK signalling in mouse astrocytes. **Bi** Similar treatments with AUY954 or CYM5541, but not UC-42-WP04, induced pERK signalling in human astrocytes. Pre-treatment with NIBR-0213 (S1PR1 antagonist) fully blocked BAF312 (100 nM)-induced pERK effects in **Aii** and **Aiii** mouse and **Bii and Biii** human astrocytes. Pre-treatment with TY52156 (S1PR3 antagonist) showed no effect on pFTY720-mediated increase of pERK in **Aiii** mouse astrocytes, while **Biii** partially attenuating pFTY720 (100 nM)-mediated effects in human astrocytes. Data presented as ±SEM (*n* = 3–6), one-way ANOVA and Newman-Keuls multiple comparison post-test compared to non-treated control, *p < 0.05, **p < 0.01, ***p < 0.001, #*p* < 0.05, ##*p* < 0.001 compared to FTY720. *R1* S1PR1, *R3* S1PR3, *R5* S1PR5, *pan* S1PR pan agonist, *R1* ^*Ant*^ S1PR1 antagonist, *R3* ^*Ant*^ S1PR3 antagonist

Given that astrocytes express S1PR1, S1PR3 and S1PR5, we examined further the role of these individual receptors to induce pERK and pAKT using selective agonists and antagonists. In mouse astrocytes, AUY954 treatment (S1PR1 agonist) induced significant pERK signalling, with a limited effect observed for CYM5541 treatment (S1PR3 agonist) (Tocris Bioscience, 4897) and no effect seen with a selective S1PR5 agonist, herein called UC-42-WP04 (compound 1 L, [7]) (Fig. 2Ai). Pretreatment with NIBR-0213 (S1PR1 antagonist) (1 μM) fully inhibited BAF312-induced pERK (Fig. 2Aii). The data also showed that pretreatment with NIBR-0213 (S1PR1 antagonist), but not TY52156 (S1PR3 antagonist), partially blocked pFTY720-mediated effects (Fig. 2Aiii). In human astrocytes, AUY954 (S1PR1 agonist) and CYM5541 (S1PR3 agonist) induced pERK signalling to a similar extent, while UC-42-WP04 (S1PR5 agonist) treatment had no effect (Fig. 2Bi). Pre-treatment with NIBR-0213 (S1PR1 antagonist) completely inhibited effects of BAF312 (Fig. 2Bii), while pre-treatment with NIBR-0213 (S1PR1 antagonist) or TY52156 (S1PR3 antagonist) modestly decreased the effects of pFTY720 on levels of pERK (Fig. 2Biii). Together, these observations suggest S1PR1 activation, and to a lesser extent, S1PR3 increases levels of pERK in mouse astrocytes. In contrast, the activation of S1PR1 or S1PR3 promotes equally pERK signalling in human astrocytes.

### Differential roles for S1PR1 and S1PR3 in pAKT signalling in mouse and human astrocytes

**Fig. 3:**
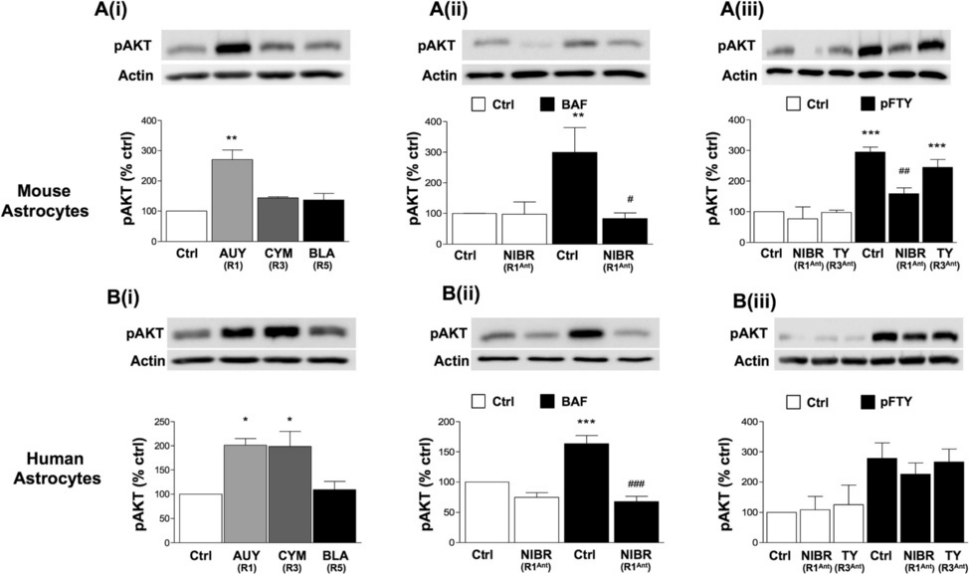
Differential roles for S1PR1 and S1PR3 in pAKT signalling in mouse and human astrocytes. Astrocytes were serum starved for 4 h before all treatments. All treatment with agonists were for 30 min and pretreatment with antagonists were at 1 μM for 1 h. **Ai** AUY954 (S1PR1 agonist, 1 μM), but not CYM5541 (S1PR3 agonist, 1 μM) or UC-42-WP04 (S1PR5 agonist, 1 μM) induced pAKT signalling in mouse astrocytes. **Bi** Similar treatments with AUY954 or CYM5541, but not UC-42-WP04, induced pAKT signalling in human astrocytes. Pre-treatment with NIBR-0213 (S1PR1 antagonist) blocked BAF312 (100nM) in **Aii** mouse and **Bii** human astrocytes. Pre-treatment with TY52156 (S1PR3 antagonist) showed no effect on pFTY720 (100 nM)-mediated increase of pAKT in **Aiii** mouse astrocytes or **Biii** human astrocytes. Data presented as ±SEM (*n* = 3–6), one-way ANOVA and Newman-Keuls multiple comparison post-test compared to non-treated control *p < 0.05, **p < 0.01, ***p < 0.001, ##*p* < 0.001 compared to FTY720. *R1* S1PR1, *R3* S1PR3, *R5* S1PR5, *pan* S1PR pan agonist, *R1* ^*Ant*^ S1PR1 antagonist, *R3* ^*Ant*^ S1PR3 antagonist

To further examine the differentially coupling of S1PRs in mouse and human astrocytes, we also examined the role of these individual receptors to induce pAKT. In mouse astrocytes, AUY954 treatment (S1PR1 agonist) induced significant pAKT signalling, with no effect observed for CYM5541 (S1PR3 agonist) or UC-42-WP04 (S1PR5 agonist) treatment (Fig. 3Ai). Pretreatment with NIBR-0213 (S1PR1 antagonist) (1 μM) fully inhibited BAF312-induced pAKT (Fig. 3). We also found that pretreatment with NIBR-0213 (S1PR1 antagonist), but not TY52156 (S1PR3 antagonist), blocked pFTY720-mediated effects (Fig. 3Aiii). In human astrocytes, AUY954 (S1PR1 agonist) and CYM5541 (S1PR3 agonist) induced pAKT signalling to a similar extent, while UC-42-WP04 (S1PR5 agonist) treatment had no effect (Fig. 3Bi). Pre-treatment with NIBR-0213 (S1PR1 antagonist) completed inhibited effects of BAF312 (Fig. 3Bii), while pre-treatment with NIBR-0213 (S1PR1 antagonist) or TY52156 (S1PR3 antagonist) did not alter the effects of pFTY720 on levels of pAKT (Fig. 3Biii). Therefore, somewhat different to pERK signalling, these results suggest that pFTY720 likely increases pAKT signalling via S1PR1 more so than S1PR3, in mouse astrocytes. In human astrocytes, similar to pERK, we find S1PR1 and S1PR3 activation equally lead to an increase in pAKT. Overall, these data suggest that S1PRs may regulate differentially the signalling pathways pERK and pAKT in mouse versus human astrocytes.

### BAF312 induces internalisation of astrocytic S1PR1

**Fig. 4:**
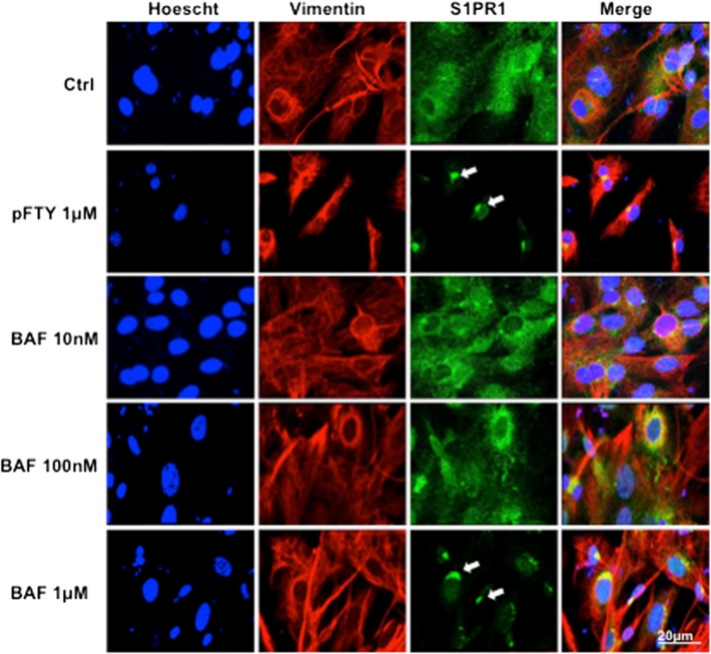
BAF312 induces internalisation of the S1PR1. Mouse astrocytes were serum starved for 4 h and then treated for 1 h with BAF312 (10 nM, 100 nM, 1 μM) or pFTY (1 μM). BAF312 at 1 μM concentration induced internalisation of S1PR1

pFTY720 is known to cause internalisation of the S1PR1 and hence is often referred to as a functional antagonist at this receptor subtype. This feature of functional antagonism forms the basis pFTY720’s efficacy in the treatment of relapsing remitting MS. Therefore, we investigated whether the S1PR1/S1PR5-selective agonist, BAF312, also induced internalisation of the S1PR1. Mouse astrocytes were serum starved and then treated for 1 h with increasing concentrations of BAF312 (10 nM, 100 nM, 1 μM or 10 μM) as well as pFTY720 (1 μM). The cells were then stained with antibodies against the astrocytic marker Vimentin and S1PR1. As previously shown [19, 22, 24], 1 μM pFTY720 induced internalisation of the S1PR1. Similarly, BAF312 induced a concentration-dependent internalisation of S1PR1 (Fig. 4). These data suggest that BAF312 may likely induce transient S1P1R stimulation followed by long-term functional antagonism similar to pFTY720.

### BAF312 induces an increase in _i_Ca^2+^ levels in human astrocytes

**Fig. 5:**
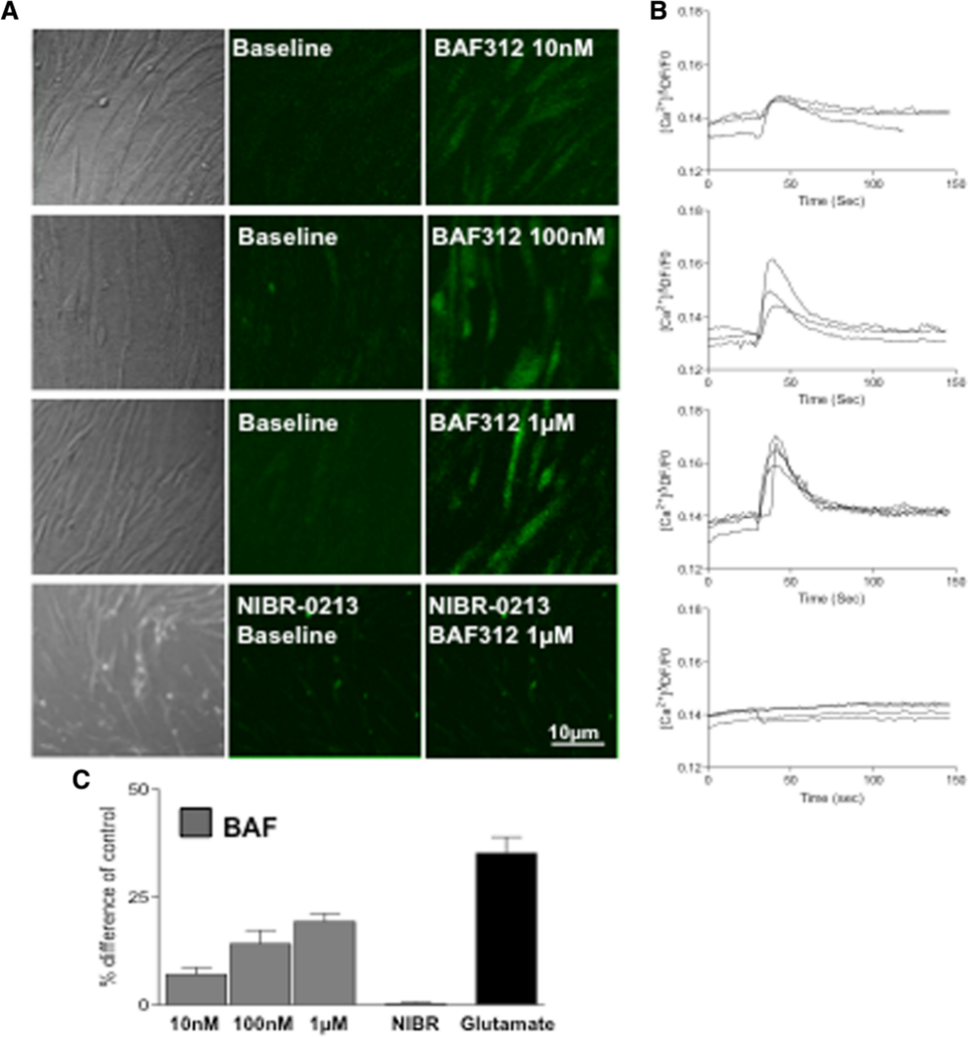
BAF312 induces an increase in _i_Ca^2+^ levels in human astrocytes. Stimulation of human astrocytes with the S1P1/S1P5 receptor agonist BAF312 increases _i_Ca^2+^ levels in Fluo-8 AM loaded human astrocytes. **a** Representative images taken from time-lapse series at baseline and after addition of BAF312 (30 s) is shown. All cells were stimulated and responded to 3 μM glutamate (150 s) (*not shown*). **b** Analysis showing traces of four separate experiments. **c** Average data showing BAF312 significantly promotes _i_Ca^2+^ levels in human astrocytes, which is attenuated by the S1PR1-selective antagonist NIBR-0213 (*n* = 4)

The endoplasmic reticulum is the major store of Ca^2+^ in astrocytes [27] and activation of S1PRs by pFTY720 and AUY954 is known to evoke Ca^2^ signalling in astrocytes [19, 22]. Here, we investigated the effect of the dual S1PR1/S1PR5 agonist BAF312 on Ca^2+^ signalling in human astrocytes. Human astrocytes were serum starved and loaded with Fluo-8 AM prior to stimulation with increasing concentrations of BAF312 (10 nM, 100 nM, and 1 μM). In these studies, BAF312 (100 nM and 1 μM) was observed to elicit an increase in _i_Ca^2+^ levels. We also noted a minority of cells showing Ca^2+^ oscillations (*data not shown*). The effect of BAF312 was abolished by pre-treatment with the S1PR1 antagonist NIBR-0213 suggesting a role for the S1PR1 subtype (Fig. 5). These studies further support our observations that the S1PR1 subtype, compared to S1PR3, plays a major role in regulating Ca^2+^ signalling in astrocytes [19, 22]. Moreover, similar to our observations for pERK and pAKT signalling pathways, these data suggest that a lack of activity on S1PR3 does not preclude BAF312 from regulating Ca^2+^ signalling.

### S1PR does not robustly attenuate levels of IL6 in astrocytes

**Fig. 6:**
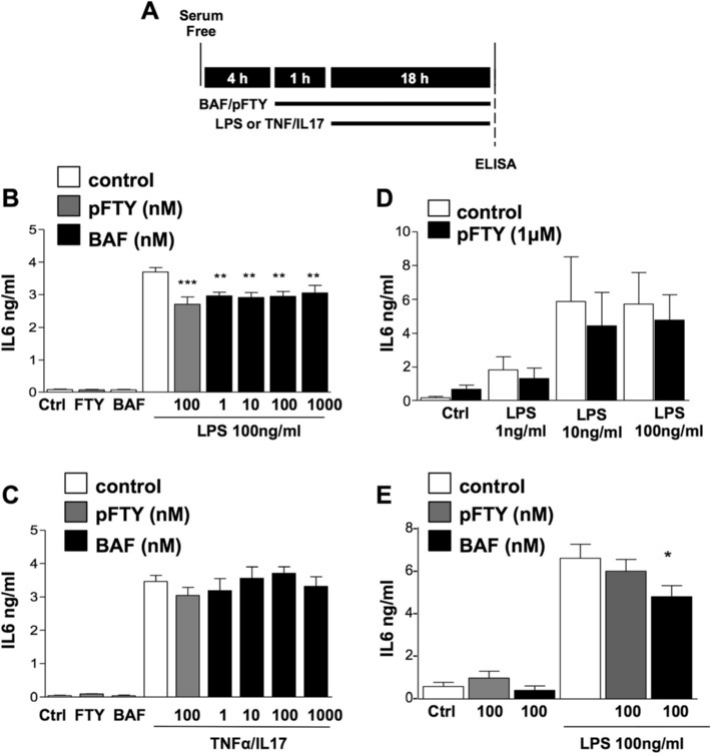
S1PR modulation selectively attenuates TLR4, but not TNFR/IL17R, mediated increase in the levels of IL6 in astrocytes. **a** Astrocytes or microglia were serum starved for 4 h, pre-treated with indicated concentrations of BAF312 and then treated with LPS (1–100 ng/ml) or TNFα/IL17. **b** Mouse astrocytes treated with LPS increased levels of IL6, which was modestly attenuated by BAF312 (*n* = 7). **c** Human astrocytes treated with LPS did not increase levels of IL6 (data not shown). In agreement with our previous studies, IL17/TNFα increased levels of IL6; however, these were not inhibited by BAF312 or FTY720 (*n* = 4). **d**, **e** Mouse microglia treated with LPS (1–100 ng/ml) increased IL6 levels in a concentration-dependent manner. pFTY720 and BAF312 moderately attenuated the LPS-induced increases in IL6. Data presented as ±SEM (*n* = 3–7), one-way ANOVA and Newman-Keuls multiple comparison post-test *p < 0.05, **p < 0.01, ***p < 0.001

Previous studies from our and other groups have demonstrated that S1PRs play a role in regulating the levels of cytokines in a number of immune cell cultures and in organotypic slice cultures [5, 9, 28, 29]. Here, we investigated the modulatory effect of BAF312 on lipopolysaccharide (LPS)- or TNFα/IL17-induced levels of the cytokine IL6 using isolated mouse and human astrocytes, respectively. Cultured human and mouse astrocytes were serum starved for 4 h and pre-treated with BAF312 (1 nM, 10 nM, 100 nM and 1 μM) for 1 h. Mouse astrocytes were then treated with LPS (100 ng/mL) for 18 h while human astrocytes were treated with TNFα/IL17 (10 ng/ml, 50 ng/ml) for 18 h, as we have previously described [20, 30]. The supernatants were then analysed by ELISA (Fig. 6a). LPS treatment of mouse astrocytes resulted in the increase of IL6 which, in our hands, was only modestly attenuated by either pFTY720 or BAF312 (Fig. 6b). Human astrocytes treated with TNFα/IL17 also resulted in the increase of IL6, which was also not noticeably altered by either pFTY720 or BAF312 treatment (Fig. 6c). We additionally investigated these effects in mouse microglia cultures and similarly found that LPS induced significant levels of IL6; however, these effects were again only modestly attenuated by pFTY720 or BAF312 (Fig. 6d, e). These data demonstrate that modulation of S1PR by BAF312 or pFTY720 does not strongly alter the levels of IL6 in astrocytes and microglia, at least in isolated cell cultures.

### BAF312 attenuates LPC-induced demyelination in mouse organotypic cerebellar slice cultures

**Fig. 7:**
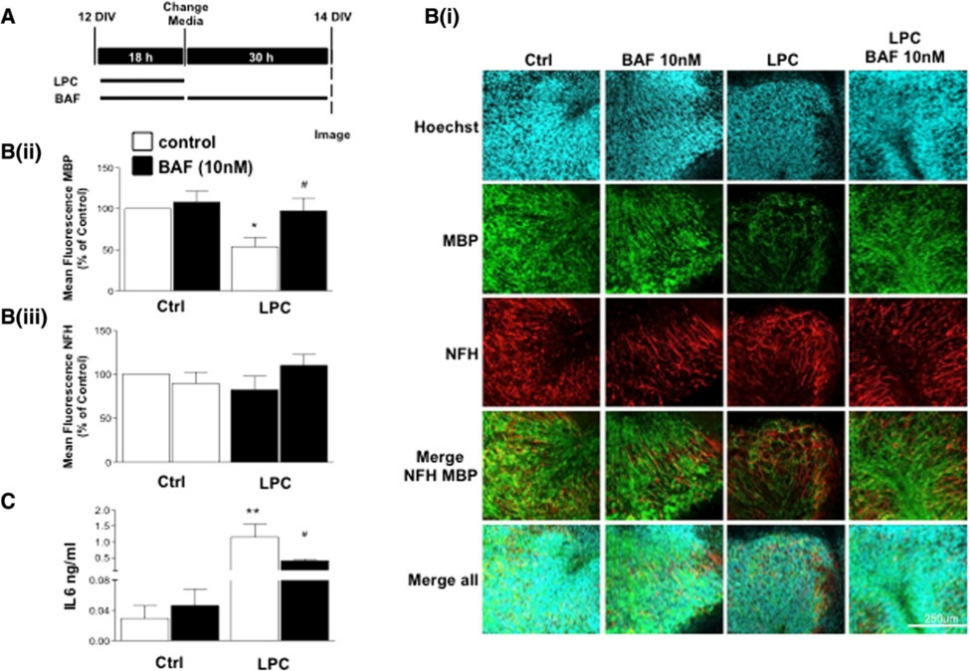
BAF312 attenuates LPC-induced demyelination and IL-6 levels in mouse organotypic cerebellar slice cultures. **A** Experimental timeline is shown, as previously described [9]. Organotypic cerebellar slice cultures were treated with LPS (0.5 mg/ml) for 18 h in the presence or absence of BAF312 and then treated with BAF213 alone for an additional 30 h (totalling 48 h) after which slices were processed for immunostaining. **Bi** Representative images show BAF312 attenuates LPC-induced demyelination as measured by MBP immunostaining, with limited effects on NFH immuoreactivity. **Bii, Biii** Data analysis of three separate experiments, demonstrating effects of BAF312 on LPC-induced changes in MBP and NFH immunostaining. **C** Organotypic cerebellar slice cultures were treated with LPS (0.5 mg/ml) for 18 h in the presence or absence of BAF312, and the media was processed for ELISA. Treatment with BAF312 attenuated the levels of IL6. Data presented as ±SEM (*n* = 3–4), one-way ANOVA and Newman-Keuls multiple comparison post-test. *p < 0.05, **p < 0.01, #*p* < 0.05 compared to LPC

pFTY720 promotes remyelination as well as limits demyelination induced by the bioactive lipid lysolecithin (lysophosphatidylcholine, LPC) [8, 9]. The high expression of S1PR5 on oligodendrocytes suggests that this receptor may also have roles in myelination. Therefore, we exposed organotypic cerebellar slice cultures to LPC (0.5 mg/ml) in the presence or absence of BAF312 (10 nM) for 18 h and treated for a further 30 h with BAF312 (10 nM) (Fig. 7a). Similar to our previous studies demonstrating the protective effects of pFTY720 [9, 21], the LPC-induced demyelination in the cerebellar slice cultures was attenuated by treatment with BAF312 (53.8 ± 11.1 % vs 97.4 ± 15.5 %) (Fig. 7b). To determine further whether BAF312 attenuated the levels IL6, the media from these organotypic slice cultures was analysed by ELISA. LPC induced at least a 60-fold increase of IL6 after 18 h (0.03 ± 0.01 ng/ml vs. 1.14 ± 0.4.ng/ml) compared with controls. Notably, in contrast to its effects in isolated astrocyte or microglia cell cultures, BAF312 (10 nM) treatment attenuated the LPC-induced levels of IL6 after 18 h (1.14 ± 0.4 ng/ml vs 0.4 ± 0.04 ng/ml), respectively (Fig. 7c). These effects were transient, where IL6 returned to high levels after 48 h (*not shown*). These findings in slice cultures may be explained by direct effects of BAF312 on immune cells rather than on astrocytes or microglia, as we have previously discussed for pFTY720 [9, 21]. Taken together, these results demonstrate that more selective S1PR1/S1PR5 compounds, such as BAF312, can significantly attenuate demyelination.

### BAF312 attenuates demyelination effects of the Krabbe disease metabolite, psychosine, in organotypic cerebellar slice cultures

**Fig. 8:**
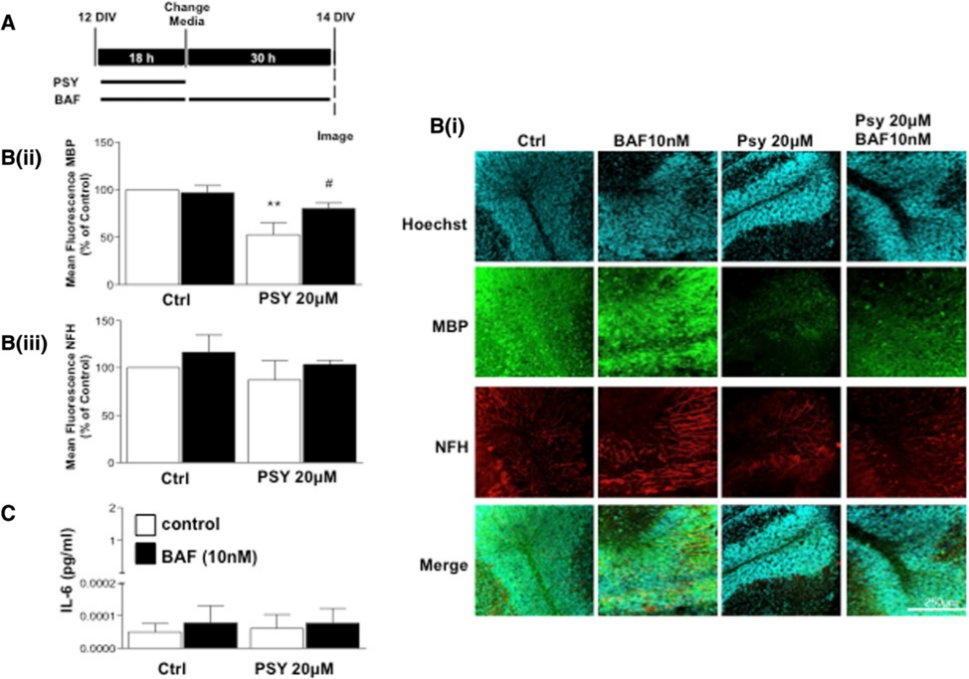
BAF312 attenuates psychosine induced demyelination in mouse organotypic cerebellar slice cultures. **A** Experimental timeline is shown. Organotypic cerebellar slice cultures were treated with psychosine (20 μM) for 18 h in the presence or absence of BAF312 and then treated with BAF213 alone for an additional 30 h (totalling 48 h) after which slices were processed for immunostaining. **Bi** Representative images show BAF312 attenuates psychosine-induced decrease in MBP immunostaining, with limited effects on NFH immunoreactivity. **Bii, Biii** Data analysis of four separate experiments, demonstrating effects of BAF312 on pyschosine-induced changes in MBP and NFH immunostaining. **C** Organotypic cerebellar slice cultures were treated with pyschosine (20 μM) for 18 h in the presence or absence of BAF312, and the media was processed for ELISA. Treatment with pyschosine, with or without BAF312, showed no changes in the levels of IL6. Data presented as ±SEM (*n* = 4)

Rapid and complete loss of myelin and the myelin-forming oligodendrocytes is one of the main pathological features of KD [31]. This illness is believed to be caused by the progressive accumulation of the toxic lipid metabolite, psychosine, in the brains of patients [31]. Recently, we have shown that pFTY720 significantly attenuates psychosine-induced demyelination in cerebellar slices [32]. Here, to corroborate these findings, we examined whether BAF312 could also attenuate psychosine-induced demyelination. Organotypic cerebellar slice cultures were exposed to psychosine (20 μM) in the presence or absence of BAF312 (10 nM) for 18 h and treated for a further 30 h with BAF312 (10 nM) (Fig. 8a). We noted that psychosine induced a modest, although not significant, decrease in the levels of neurofilament H (NFH). More importantly, psychosine induced significant demyelination, as measured by the loss of MBP staining, which was significantly attenuated by BAF312 (10 nM) (52.7 ± 12.5 % vs 80.3 ± 5.7 %) (Fig. 8b). We again measured the levels of IL6 by ELISA in these experiments, after the initial 18-h treatment. In this case, unlike LPC, psychosine did not induce an increase in the levels of IL6 (Fig. 8c). We suggest, therefore, that the modulation of S1PR1/S1PR5 by BAF312 is sufficient to rescue demyelination associated with or without changes in cytokines induced by toxins such as LPC and psychosine, respectively.

## Discussion

### Summary of findings

In this study, we investigated the role S1PR1 and S1PR5 in astrocyte function by using the S1PR3-sparing drug BAF312 (Siponimod), which is currently in phase II clinical trials for secondary progressive MS. Similar to pFTY720 [19, 24], we found BAF312 induced levels of pERK, as well as pAKT, in a concentration-dependent manner in both human and mouse astrocytes. Furthermore, we demonstrated that the selective S1PR1 agonist, AUY954, induced pERK and pAKT signalling in both human and mouse astrocytes, the S1PR3 agonist, CYM5541, induced pERK and pAKT only in human astrocytes and the S1PR5 agonist, UC-42-WP04, did not induce ERK or AKT phosphorylation in either mouse or human astrocytes. The S1PR1 specific antagonist, NIBR-0213 [23], inhibited the effects of BAF312 on pERK and pAKT signalling in human and mouse astrocytes, suggesting that BAF312-induced pERK and pAKT is mediated primarily via the S1PR1 subtype. Using the S1PR1 and S1PR3 antagonists, NIBR-0213 and TY52156, we showed that S1PR1 played a more critical role in regulating pAKT signalling compared to pERK in mouse astrocytes and that both S1PR1 and S1PR3 played similar roles in pERK and pAKT signalling in human astrocytes. BAF312 also stimulated levels of Ca^2+^ in human astrocytes, which was attenuated by NIBR-0213, indicating further an importance of the S1PR1 subtype. Similar to pFTY720, the treatment of astrocytes with BAF312 induced S1PR1 internalisation in a concentration-dependent manner. In our hands, neither pFTY720 nor BAF312 greatly attenuated the release of IL6 from LPS- or TNFα/IL17-stimulated mouse and human astrocytes, respectively. In addition, pFTY720 did not attenuate IL6 levels from LPS-stimulated mouse microglial cultures. We also report here that BAF312 attenuated both LPC- and psychosine-induced demyelination in organotypic slice cultures. The effects of BAF312 in LPC-induced demyelination was accompanied by a decrease in the induced levels of IL6, in agreement with our previous study [9]. Of most interest, the effect of BAF312 in the LPC and psychosine-induced demyelination was not associated with changes in the levels of IL6. Overall, these studies suggest that more selective S1PR compounds such as BAF312 may regulate myelination state in both inflammatory and non-inflammatory models of demyelination.

### Differential coupling of S1PR subtypes to pERK and pAKT in mouse and human astrocytes.

The signalling molecules pERK and pAKT are both associated with activating pro-survival pathways, and S1PRs are known to modulating these pathways [33–35]. To further delineate the relative contributions of S1PR1, 3 or 5 to modulate ERK and AKT phosphorylation in astrocytes, we used the selective agonists AUY954 (S1PR1), CYM5541 (S1PR3) and UC-42-WP04 (S1PR5) to stimulate human and mouse astrocytes. We found that S1PR5 is not coupled to pERK and pAKT in mouse or human astrocytes. In contrast, the S1PR1 agonist, AUY954, induced ERK and AKT phosphorylation in both human and mouse astrocytes indicating that S1PR1 plays a central role in the induction of pERK and pAKT. Interestingly, the S1PR3 agonist, CYM5541, induced ERK and AKT phosphorylation only in human astrocytes suggesting that S1PR3 may have a greater influence on pERK and pAKT signalling in human astrocytes in comparison to mouse astrocytes. By use of the S1PR1 antagonist, NIBR-0213 and the S1PR3 antagonist, TY52156, we also demonstrated the following rank order of importance for pFTY720 activation of pERK and pAKT in human and mouse astrocytes: S1PR1 > S1PR3 for pERK signalling and S1PR1 >> S1PR3 for pAKT signalling in mouse astrocytes; S1PR3 ≥ S1PR1 for pERK signalling and S1PR1 = S1PR3 for pAKT signalling in human astrocytes. Such species-dependent differences between human and mouse S1PR1 and S1PR3 function has been reported before. For example, a variance in S1PR1 and S1PR3 function between human and mouse has been seen previously as the initial bradycardia experienced after pFTY720/BAF312 treatment is reported to be species-dependent [10, 16]. In addition, bone marrow-derived mesenchymal stem cells (MSCs) have been shown to express collagen via the activation of S1PR1 and S1PR3 in mouse cells, while collagen expression MSCs is negatively regulated by S1PR1 and S1PR3 in human cells (Yang et al. [36]). In summary, our results suggest that S1PR1, compared to S1PR3, plays a more dominant role in pERK/pAKT signalling in mouse astrocytes, whereas S1PR1 and S1PR3 are equally important in regulating these pathways human astrocytes.

### The role of S1PRs in regulating levels of IL6 in microglia and astrocytes

Specific knockout of S1PR1 from astrocytes or pFTY720 treatment reduces the levels of IL1β, IL6 and IL17, which are cytokines increased during the course of EAE [5]. In agreement, pFTY720 attenuates the levels of cytokines such as IL6, TNFα and IL1β from LPS-activated microglia, in addition to enhancing the levels of brain-derived nerve factor (BDNF) and glial-derived nerve factor (GDNF) [37]. Treatment of organotypic cerebellar slice cultures with LPC for 2 days also increases the number of both Iba1+ and GFAP+ cells, which subsides after 14 days of LPC treatment, suggesting a temporal effect of LPC on microglia and astrocyte cells numbers [8]. The treatment of these slice cultures with pFTY720 alone does not appear to alter astrocyte levels [9], but treatment with pFTY720 after a 2-day exposure to LPC increases the number of Iba1+ and GFAP+ cells, compared to LPC alone [8]. Importantly, both treatment with pFTY720 at the same time as LPC [9] and 2 days after LPC [8] shows increased levels of myelination compared to LPC alone, suggesting that pFTY720 can likely inhibit processes of demyelination and promote events enhancing remyelination. Here, we showed that while BAF312 and pFTY720 modestly attenuate the levels of IL6 from LPS-stimulated mouse astrocytes, they had no observable effect on TNFα/IL17 stimulated human astrocytes. We also found that pFTY720 did not strongly attenuate LPS-induced IL6 in cultured mouse microglia. In contrast, BAF312 attenuated the levels of IL6 levels in organotypic slice cultures treated with LPC, similar to our previous data [9]. Unlike isolated cell cultures, organotypic slice cultures preserve the brain architecture and also contain immune cells [9, 38, 39]. Thus, one possible explanation for our findings using cultured astrocytes and microglia, versus organotypic slice cultures, may be the absence or presence of peripheral immune cells, respectively. It remains unclear, however, the number(s) or type(s) of immune cells in these slice cultures that are required to confer a BAF312 effect, if any. A small and undetectable number of immune cells in these slices may indeed be sufficient to trigger a subsequent amplified response by glial cells. The idea that immune cells can induce demyelination and that S1PR drugs can attenuate this response is in line with our previous findings demonstrating that MOG-reactive splenocytes can induce demyelination when added to organotypic slice cultures and their treatment with pFTY720 reverses this effect [21].

### The potential use of S1PRs as drug targets in Krabbe disease

The treatment of organotypic slices with the demyelinating agent LPC is an established in vitro model to study the effects demyelination, where drugs such as pFTY720 have shown to be protective [8, 9]. In the present study, we found that BAF312 attenuated both LPC-induced demyelination as well as the levels of IL6 in organotypic slice cultures, similar to that observed for pFTY720 [32]. We also used the toxic agent psychosine to induce demyelination, which accumulates in the brain of patients with KD causing widespread demyelination and almost complete loss of oligodendrocytes in the white matter [31]. Notably, we have found that psychosine induces demyelination in a manner that is not associated with altered levels of cytokines, including IL1β, TNFα and IL6 [32]. Psychosine induced demyelination that was not associated with altered levels of IL6 as we have previously observed [32], and most importantly, BAF312 attenuated this psychosine-induced demyelination. Overall, these studies suggest that S1PR modulation is protective in both inflammatory and non-inflammatory models of demyelination.

### Conclusions

The clinical success of pFTY720 in the treatment of relapsing remitting MS demonstrated the therapeutic potential of S1PR modulation and prompted efforts to develop more specific agonists for these receptors. Modulators with selectivity towards S1PR1 and S1PR5 seemed of particular interest as preclinical observations in mice suggested that S1PR3 may be responsible for the transient bradycardia experienced after the first dose of pFTY720 [10, 15]. Furthermore, S1PR1 modulation plays a key role in lymphocyte migration [40] and S1PR5 is expressed on oligodendrocytes where it plays a role in myelination [7, 8, 13]. This has led to the synthesis of the dual S1PR1/S1PR5 agonist: BAF312 (Siponimod). Similar to pFTY720, BAF312 has been reported to supress pre-clinical symptoms in animal models of EAE. This drug also selectively decreases T cells (CD4^+^, naive and central memory) and B cells in healthy human volunteers, while sparing effector memory T cells [16]. In a phase II clinical study for relapsing remitting MS, BAF312 reduced brain MRI lesions up to 80 % in comparison with placebo control [18]. Bradycardia is also observed with BAF312, but can be mitigated with a dose titration regimen [17], thus providing additional benefits. Importantly, BAF312 is currently undergoing a phase III trial to investigate its efficacy in patients with the chronic secondary progressive MS, a disease in which there is a particular lack of treatments [41]. Given that pFTY720 has shown limited beneficial effects in patients with the chronic primary progressive MS (ClinicalTrials.gov:NCT00731692) and limited effect in secondary progressive EAE [42], the outcome of these clinical trials with BAF312 are highly awaited. In addition to the differences between primary and secondary progressive MS, differential effects of the two drugs on human astrocytes, which are not observed in mouse astrocytes, might influence the clinical outcome of the two drugs in progressive MS.

Here, we demonstrated that BAF312 regulates a number of signalling pathways in human and mouse astrocytes. In addition, this compound attenuated demyelination in organotypic slice cultures induced by LPC and by psychosine, a toxic metabolite that accumulates in the brains of patients with KD. These findings suggest that the S1PR drugs such as BAF312 may have utility beyond MS, in a range of demyelinating diseases such as KD, for which no therapies currently exist.

## Abbreviations

BDNF: brain-derived nerve factor
CNS: central nervous system
EAE: experimental autoimmune encephalomyelitis
GDNF: glial-derived nerve factor, central nervous system
KD: Krabbe disease
LPS: lipopolysaccharide
MS: multiple sclerosis
pFTY720: phosphorylated FTY720
S1PR: spingosine-1-phosphate receptors
